# Prevalence of chromosome 8p11.2 translocations and correlation with myeloid and lymphoid neoplasms associated with *FGFR1* abnormalities in a consecutive cohort from nine institutions in Japan

**DOI:** 10.1007/s12185-024-03740-0

**Published:** 2024-03-08

**Authors:** Kensuke Usuki, Takuro Kameda, Noriaki Kawano, Tomoki Ito, Yoshinori Hashimoto, Kotaro Shide, Hiroshi Kawano, Masaaki Sekine, Takanori Toyama, Hiromitsu Iizuka, Seiichi Sato, Masanori Takeuchi, Junzo Ishizaki, Kouichi Maeda, Michikazu Nakai, Kiyoshi Yamashita, Yoko Kubuki, Kazuya Shimoda

**Affiliations:** 1 Department of Hematology, NTT Medical Center Tokyo, Tokyo, Japan; 2 Division of Hematology, Diabetes, and Endocrinology, Department of Internal Medicine, Faculty of Medicine, University of Miyazaki, Miyazaki, Japan; 3 Miyazaki Prefectural Miyazaki Hospital, Miyazaki, Japan; 4 First Department of Internal Medicine, Kansai Medical University, Osaka, Japan; 5 Department of Hematology, Tottori Prefectural Central Hospital, Tottori, Japan; 6 Koga General Hospital, Miyazaki, Japan; 7 Miyazaki Prefectural Nobeoka Hospital, Nobeoka, Japan; 8 Miyakonojo Medical Center, Miyakonojo, Japan; 9 Aisenkai Nichinan Hospital, Nichinan, Japan; 10 Clinical Research Support Center, University of Miyazaki Hospital, Miyazaki, Japan

**Keywords:** 8p11, FGFR1, Eosinophilia

## Abstract

Myeloid and lymphoid neoplasms associated with *FGFR1* abnormalities (MLN-*FGFR1* abnormalities) are rare hematologic malignancies associated with chromosome 8p11.2 abnormalities. Translocations of 8p11.2 were detected in 10 of 17,039 (0.06%) unique patient cytogenetic studies performed at nine institutions in Japan. No inversions or insertions of 8p11.2 were detected. Among the 10 patients with 8p11.2 translocations, three patients were diagnosed with MLN-*FGFR1* abnormalities, which were confirmed by FISH analysis. Peripheral blood eosinophilia was observed in all three patients, and all progressed to AML or T-lymphoblastic lymphoma/leukemia. The prevalence of 8p11.2 translocations in clinical practice and the proportion of MLN-*FGFR1* abnormalities in patients with 8p11.2 translocations in Japan were consistent with those in previous reports from Western countries.

## Introduction

Chromosomal 8p11 abnormalities with Fibrocyte growth factor receptor 1 (*FGFR1)* rearrangement is a rare hematopoietic disorder with widespread clinical and pathologic features, including myeloproliferative neoplasms (MPN), myelodysplastic syndrome (MDS), and MPN/MDS, often associated with eosinophilia, lymphadenopathy, usually T-lymphoblastic lymphoma/leukemia (T-LBL), and progression to acute myeloid leukemia (AML) [1]. The disease progresses rapidly, usually to acute leukemia within one year, and the prognosis is poor. In the 5th WHO classification of hematopoietic and lymphoid neoplasms, it was classified as “myeloid and lymphoid neoplasms associated with *FGFR1* abnormalities (MLN-*FGFR1* abnormalities)” in the “myeloid and lymphoid neoplasms with eosinophilia and tyrosine kinase gene fusions (MLN-TK)” [2].

FGFR1 is a member of the receptor tyrosine kinase superfamily, and the molecular pathogenesis of MLN-*FGFR1* abnormalities is characterized by the generation of fusion proteins with an intact kinase domain of FGFR1 [3]. Currently, it has been reported that 17 *FGFR1* gene rearrangements exist in MLN-*FGFR1* abnormalities, including 15 translocations, one insertion, and one inversion [1]. The difference of partner genes with *FGFR1* rearrangement is thought to be the cause of the variety of phenotypes of MLN-*FGFR1* abnormalities, which makes it difficult to list MLN-*FGFR1* abnormalities as a differential diagnosis. Chromosomal analysis is one of the standard examinations initially performed for the diagnosis of hematologic malignancies, and the chromosomal abnormalities involving 8p11.2 are one of the initial keys for the diagnosis of MLN-*FGFR1* abnormalities. The purpose of this study is to describe the prevalence of 8p11.2 abnormalities in Japanese hematology practices.

## Materials and methods

Cytogenetic analysis was performed as part of routine patient care at each institution. Twenty metaphases were analyzed per patient, and karyotypes were described according to the International System for Human Cytogenomic Nomenclature. Fluorescence in-situ hybridization (FISH) analysis of interphase nuclei was performed using dual-color split probes for the 8p11.2 loci (Leica Biosystems Inc., Nußloch, Germany).

The cytogenetic database from 2010 to 2022 in nine institutions was screened for 8p11.2 translocations, inversions, and insertions. For patients with 8p11.2 abnormalities, medical records were reviewed to collect data including clinical diagnosis, peripheral blood analysis, and their clinical course.

The study was carried out following the guidelines for the ethical guidelines for medical and health research involving human subjects and approved by the institutional review boards at each participating institution.

## Statistical analysis

The required sample size was calculated as follows: n ≧ (z^2 * p * (1-p)) / (e^2), where n is the sample size, z is the z-score, p is the population proportion, and e is the margin of error. Values of 95% and 0.04% were used for z and e, respectively. If the prevalence of 8p11.2 translocations in Japan is 0.06%, which is similar to that in Western countries, the required sample size is 13,397.

## Results

From 2010 to 2022, 17,039 unique patient cytogenetic studies were performed in nine institutions. Among them, chromosome 8p11.2 translocations were identified in 10 patients (0.06%). There are no cases of inversion or insertion of 8p11.2.

**Fig. 1:**
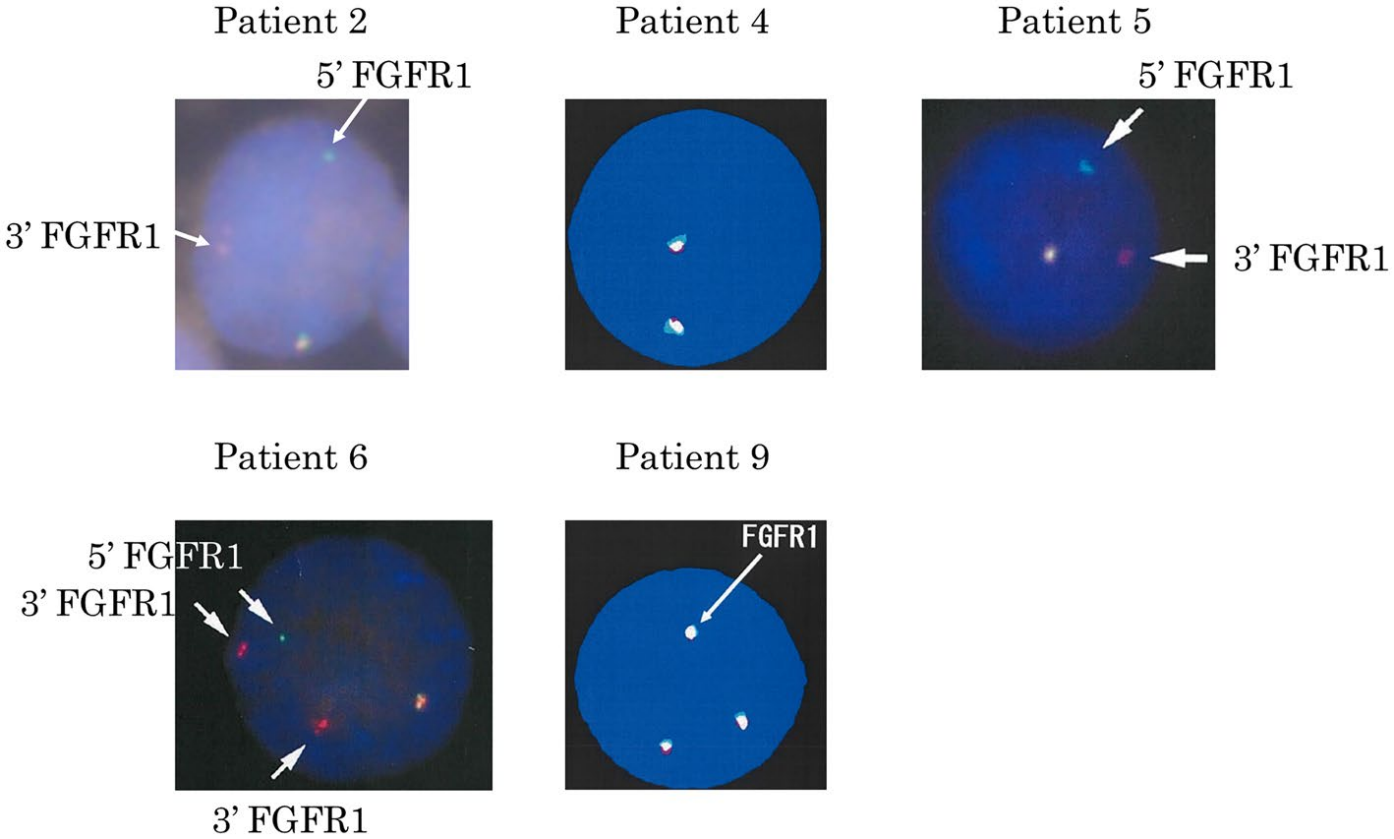
***FGFR1* cleavage FISH on interphase nuclei.** 5ʹ *FGFR1* FISH probe was labeled green and 3ʹ *FGFR1* FISH probe was labeled red. Fusion signal (red/green or yellow, white) indicates unaffected *FGFR1*. Splitting of the fusion signal (separated red or green signal) marks the rearrangement involving the *FGFR1* locus. A split *FGFR1* signal was detected in patient 2, patient 5, and patient 6. Amplification of the *FGFR1* signal was detected in case 9

Among the 10 patients with 8p11.2 translocations, three patients (patient 2, patient 5, and patient 6) showed typical clinical manifestations of MLN-*FGFR1* abnormalities (Table 1). Patient 2 was previously reported as a case report [4]. He was initially diagnosed with angioimmunoblastic T-cell lymphoma (AITL) associated with eosinophilia and achieved a first complete remission (CR) after treatment with cyclophosphamide, doxorubicin, vincristine, and prednisone (CHOP). His AITL relapsed four years later and he underwent CHASE therapy (cyclophosphamide, cytosine arabinoside, etoposide, and dexamethasone), followed by autologous peripheral blood stem cell transplantation. The second CR status lasted for approximately one year. MDS/MPN features appeared in BM samples and AML occurred. Patient 5 initially had features of atypical chronic myeloid leukemia (aCML). Two years later, she developed lymphoadenopathy and eosinophilia and was diagnosed with T-lymphoblastic lymphoma (LBL). The third patient (patient 6) was diagnosed with MLN with *FGFR1* rearrangement accompanied by eosinophilia. AML developed six months later. FISH studies demonstrated disruption of *FGFR1* gene regions in all three patients (Fig. 1). Eosinophilia in the peripheral blood was observed in all three cases with disruption of *FGFR1* gene regions. Dysplasia in the eosinophils was not observed in patients 5 and 6; however, hypogranular eosinophils were detected in patient 2.

**Table 1:** Characteristics of patients with 8p11.2 translocations

Patient number	Age/Sex	Karyotypes	FISH	Diagnosis associated with 8p11.2 translocation	Absolute cell count (× 10^9^/L)		Antecedent disease	Subsequent transformation
					Eosinophils	Monocyte		
1	70/F	46, XX, t(8;15)(p11.2;q11.2), t(14;18)(q32;q21) [2]	n.d	Follicular lymphoma grade 3A	0.2	0.26		
2	61/M	46, XY, t(8;13)(p11.2;q12) [4] 46, XY [16]	split signal	AITL	2.8	1.9		MDS/MPN, AML
3	48/F	46, XX, t(8;16)(p11.2;p13.3) [18] 46, XX [2]	n.d	AML M5b	0.2	0.28		
4	69/F	46, XX, t(1;7;8)(p13;q32;p11.2), t(6;9)(p21;p24), add(13)(q32) [4] 46, XX, idem, add(4)(p11) [7] 46, XX [9]	no split signal	DLBCL	0.09	0.62		
5	70/F	46, XX, t(8;13)(p11.2;q12) [18] 46, XX [2]	split signal	T-LBL	19.9	2.3	aCML	
6	55/M	48, XY, t(8;13)(p11.2;q12), + der(13)t(8;13), + 21 [17]	split signal	MLN-FGFR1 translocation, T-lymphoblastic leukemia/lymphoma	5.2	4.6		AML
7	73/M	59, XY, -X, dic (1;8)(p13;p11.2), -6, add(7)(p15), -8, -10, -12, -13, add(14)(q32), + add(15)(q22),-16, -17, -18, + 19, -20, -22 [2] 59, XY, -X, dic (1;8)(p13;p11.2), -6, add(7)(p15), -8, -10, -12, -13, + add(15)(q22), -16, -17, -18, + 19, -20, -21, -22, + mar [2] 58, XY, -X, dic (1;8)(p13;p11.2), -6, add(7)(p15), -8, -10, -12, -13, + add(15)(q22), -16, -17, -18, -18, + 19, -20, -21, -22, + mar [1] 58, XY, -X, dic (1;8)(p13;p11.2), -6, add(7)(p15), -8, -10, -12, -13, + add(15)(q22), -16, -17, -18, + 19, -20, -21, -22 [1] 46, XY [14]	n.d	Multiple myeloma	0.1	3.1		
8	72/M	46, XY, der(8)t(1;8)(q12;p11.2), t(10;14)(p11.2;q32) [2] 46, idem, -16, + 22 [1] 46, XY [17]	n.d	NHL, marginal zone lymphoma	0.08	0.16		
9	53/F	43, XX, der(3)t(1;3)(q25;p13),-7, dic(8;?)(p11.2;?), -15, -18 [8] 61, idem, + 1, + 2. + 2, + 3, + 6, + 6, + 7, + 8, + i(8)(q10), + 14, + 14, + 15, + 16, + 18, + 19, + 19, + 20, + 20, + 21 [3]	3 signals	Acute erythroblast leukemia	0.06	0.7	MDS	
10	59/M	46, XY, idic(8)(p11.2), t(9;22)(q34;q11.2) [20]	n.d	Ph-positive ALL	0	0.03		

Abbreviations: FISH, fluorescence in situ hybridization; N.D., not done; AITL, angioimmunoblastic T-cell lymphoma; AML, acute myeloid leukemia; DLBCL, diffuse large B-cell lymphoma; T-LBL, T-cell lymphoblastic lymphoma/lymphoma; MLN-*FGFR1*, myeloid and lymphoid neoplasms associated with *FGFR1* abnormalities; NHL, non-Hodgkin’s lymphoma; Ph, Philadelphia chromosome; ALL, acute lymphoblastic leukemia; aCML, atypical chronic myeloid leukemia; MDS, myelodysplastic syndrome

The other seven patients did not show typical features of MLN with *FGFR1* rearrangement. Among them, patient 3 was diagnosed as AML M5b with t(8;16)(p11.2;p13.3). Patient 9 was diagnosed with MDS, and two years later, she developed AML with severe hemophagocytosis. The other five patients were clinically diagnosed with lymphoid malignancies, including follicular lymphoma, diffuse large-cell B-cell lymphoma, multiple myeloma, marginal zone lymphoma, and Philadelphia chromosome-positive ALL. FISH analysis was performed in two patients (patient 4 and patient 9). No disruption of the *FGFR1* gene was observed in patient 4, and the *FGFR1* gene was amplified in patient 9 (Fig. 1). In addition, there were 15 cases with eosinophilia that underwent FISH examination using dual-color split probes for the 8p11.2 loci before routine chromosomal analysis, and no case showed disruption of *FGFR1* gene regions.

To examine whether there is a regional bias in the frequency of the subtypes of hematological diseases in this study, we compared the patient number of major hematological diseases between the Japanese Society of Hematology (JSH) registry data ( http://www.jshem.or.jp/modules/member/index.php?content_id=29) and seven institutions from this study. These data were unavailable for the remaining two institutions. The disease distribution is almost similar between JSH registry data and our study, however, proportion test revealed that the proportions of AML and malignant lymphoma are lower, and those of MDS, MPN, and immune thrombocytopenic purpura are higher in our study compared to JSH registry data.

## Discussion

We showed that the prevalence of chromosome 8p11.2 abnormality in Japanese clinical practice was 0.06%, which is similar to that reported by Mayo Hospital of 0.06%; suggesting that there is no ethnical difference in the frequency of 8p11.2 abnormalities in hematological diseases [5]. In addition, not all patients with 8p11.2 abnormalities were diagnosed with MLN-*FGFR1* abnormalities. About one third of them were categorized as MLN-*FGFR1* abnormalities in our study, which was also consistent with a previous report in which four of 14 patients with 8p11.2 translocations and in another report, four of 12 patients with 8p11.2 translocations were diagnosed as MLN-*FGFR1* abnormalities [5, 6].

Although the prevalence of chromosome 8p11.2 abnormalities in Japan was similar to that in Western countries, the disease types were different. The majority of Japanese patients with 8p11.2 abnormalities had lymphoid malignancies, whereas those in Western countries had myeloid malignancies. However, we cannot find a reasonable explanation for this discrepancy.

All three cases with MLN-*FGFR1* abnormalities in this report harbored t(8;13)(p11.2;q12), which is the most common chromosomal abnormality in MLN-FGFR1 abnormalities [1]. In this translocation, the partner gene with *FGFR1* is *ZNF198* (previously reported as *ZMYM2*, *FIM*, and *RAMP*) located at 13q11-12, and the zinc finger domain of ZNF198 is fused to the tyrosine kinase domain of FGFR1. Recipient mice transplanted with bone marrow cells transfected with *ZMYM2::FGFR1* developed myeloproliferation and intestinal T-cell lymphoma, and in immunodeficient mice, human cord blood CD34^+^ cells transfected with *ZMYM2::FGFR1* showed expansion of multiple myeloid cell lineages and accumulation of blasts in BM [7, 8]. In patients with t(8;13)(p11.2;q12), lymphadenopathy and hepatosplenomegaly are often the first symptoms, and most patients are diagnosed with T-LBL/T-lymphoma [1]. Consistent with this, all three patients in our study developed T-cell malignancies in their clinical course.

Umino et al. reported the characteristics and prognosis of 45 cases with MLN-*FGFR1* abnormalities collected from a computerized search of the medical literature using PubMed® [9]. At diagnosis, 31% and 69% of patients were in chronic phase as MPN or MDS and in advanced phase as acute leukemia or lymphoblastic lymphoma, respectively. Approximately half of patients in chronic phase transformed to advanced blast phase within one year, and the median OS from diagnosis was 9 months if allogeneic HSCT was not performed before transformation to advanced blast phase. The OS for patients in advanced phase was dismal, and the 1-year OS was 29.8%. Since *FGFR1* rearrangement leads to constitutive activation of tyrosine kinase, which induces abnormal proliferation, inhibitors targeting FGFR1 kinase activity have been developed [10, 11]. Among them, pemigatinib, a reversible ATP-competitive FGFR inhibitor, has been recently approved as a novel drug for treatment of myeloid/lymphoid neoplasms with *FGFR1* rearrangement in the US and Japan. To select appropriate patients for pemigatinib therapy, FISH analysis demonstrating *FGFR1* gene disruption would be desirable in addition to chromosomal 8p11.2 abnormalities for patients who do not exhibit the typical clinical features of MLN-*FGFR1* abnormalities, because approximately one third of patients with 8p11.2 abnormalities were diagnosed as MLN-*FGFR1* abnormalities in our study and previous reports [5, 6].

Of other seven patients without typical features of MLN with *FGFR1* rearrangement, one patient was diagnosed as AML M5b with t(8;16)(p11.2;p13.3). *MYST3* (MYST histone acetyltransferase 3) is disrupted by the chromosomal 8p11.2 translocation, as is *FGFR1*, and the most common translocation partner gene for *MYST3* is *CEBBP* (CREB-binding protein), located on chromosomal 16p13.3 [12]. As the chromosomal translocation t(8;16)(p11.2;p13.3) is reported to be associated with an aggressive form of AML M4/M5, the 8p11.2 abnormality detected in this patient is speculated to be a *MYST3*-*CREBBP* rearrangement, although we cannot show it by FISH analysis due to lack of residual BM samples [13]. Another patient whose MDS progressed to AML had *FGFR1* amplification. The overexpression or amplification of *FGFR1* has been reported in 10–20% of breast cancer, head and neck squamous cell carcinoma, and lung squamous cell carcinoma, and its association with poor survival has been reported in breast cancer, head and neck squamous cell carcinoma, but not in lung cancer [14–16]. In contrast, overexpression or amplification of *FGFR1* was rare in hematological malignancies. For the remaining four patients (patients 1, 7, 8, and 10), FISH examination using dual-color split probes for the 8p11.2 loci was not performed because of the lack of residual samples; therefore, it is difficult to completely exclude them from having MLN with *FGFR1* rearrangement. The breakpoint of partner genes rearranged with 8p11.2, in these four cases included 1p13, 1q12, 8p11.2, and 15q11.2. As these chromosomal breakpoints have not been previously reported to generate *FGFR1* fusion genes and cause MLN with *FGFR1* rearrangement [1], the possibility of their diagnosis as MLN with *FGFR1* rearrangement might be low, along with the absence of the typical clinicopathological features of MLN with *FGFR1* rearrangement.

In conclusion, the prevalence of 8p11.2 abnormalities in Japanese clinical practice and the proportion of MLN-*FGFR1* abnormalities among them were almost consistent with those in previous reports from Western countries.
